# Parasporin A13-2 of *Bacillus thuringiensis* Isolates from the Papaloapan Region (Mexico) Induce a Cytotoxic Effect by Late Apoptosis against Breast Cancer Cells

**DOI:** 10.3390/toxins13070476

**Published:** 2021-07-09

**Authors:** Diego Becker Borin, Karen Castrejón-Arroyo, Alain Cruz-Nolasco, Miguel Peña-Rico, Michele Rorato Sagrillo, Roberto C. V. Santos, Lucas Silva de Baco, Lemuel Pérez-Picaso, Luz Camacho, A. Karin Navarro-Mtz

**Affiliations:** 1 Instituto de Biotecnología, Universidad del Papaloapan, Tuxtepec, Oaxaca 68301, Mexico; dbborin@gmail.com (D.B.B.); mapena@unpa.edu.mx (M.P.-R.); 2 División de Estudios de Posgrado, Universidad del Papaloapan, Tuxtepec, Oaxaca 68301, Mexico; ing.castrejon_k@hotmail.com (K.C.-A.); alandurst13@hotmail.com (A.C.-N.); 3 Graduate Program in Nanoscience, Franciscan University, Santa Maria, Rio Grande do Sul 97010-032, Brazil; sagrillorm18@gmail.com; 4 Oral Microbiology Laboratory, Universidade Federal de Santa Maria, Santa Maria, Rio Grande do Sul 97065-060, Brazil; robertochrist@gmail.com; 5 Oncologia, Franciscan University, Santa Maria, Rio Grande do Sul 97015-450, Brazil; lucasbaco2014@gmail.com; 6 Instituto de Química Aplicada, Universidad del Papaloapan, Tuxtepec, Oaxaca 68301, Mexico; lemuelp@unpa.edu.mx; 7 Laboratorio de Nutrición Experimental, Instituto Nacional de Pediatría, Ciudad de México 4530, Mexico; camacho.luz@gmail.com

**Keywords:** parasporins, anticancer, cytotoxicity, late apoptosis, MCF-7

## Abstract

The protein A13-2 was obtained from *Bacillus thuringiensis* strains isolated from the Papaloapan watershed region (Oaxaca, Mexico). The cytotoxic activity of parasporal inclusions was studied against breast cancer cell line (MCF-7) and normal cell (human peripheral blood mononuclear cells). The MTT, the formation of reactive species, nitric oxide, free cell DNA, and the type of death cellular were assessed. The protein A13-2 shows the highest cytotoxic activity against MCF-7 (13% cell viability at 6 µg/mL), the extracellular DNA increases, and it shows no stress for reactive species or nitric oxide. Besides, the A13-2 parasporin shows no toxicity to peripheral blood mononuclear cells, and it does not generate changes in nitric oxide levels or free cell DNA. Due to that, the cytotoxic effect of A13-2 was specific for MCF-7, and it does not affect normal cells. According to microscopy and flow cytometry, A13-2 parasporin leads to the death of MCF-7 cells by late apoptosis together with necrosis and without allowing the triggering of the survival mechanisms. When analyzed together, our results show for the first time that the A13-2 protein isolated from Mexican strains of *B. thuringiensis* preferentially kills MCF- 7 (cancer cells) over HEK 293 and PBMC cell lines (normal cells), thus representing a promising alternative for the treatment of cancer breast.

## 1. Introduction

*Bacillus thuringiensis* is a Gram-positive bacterium that produces parasporal crystals during its sporulation stage [1]. The parasporal crystals can be constituted of the proteins Cry, Cyt, and parasporins, each with particular activities—insecticidal, hemolytic, and anticancer, respectively [2,3]. Parasporins are a set of proteins that are divided or grouped into six families (PS1-PS6) with different sizes and modes of action [4]. It has been demonstrated that parasporins are cytotoxic against mammalian cancer cells [5,6,7,8]. The molecular weights of proteins are from 30 to 94 kDa. Most *B. thuringiensis* parasporins were obtained from strains isolated on the Asian continent (Japan, Vietnam, Malaysia, and India) [9], although, there are also reports of this type of strain in America, in the Caribbean islands, Canada and México [9,10].

The parasporins are good candidates for cancer therapy due to their selective activity against cancer cells and their ubiquity [11]. Breast cancer is the second most prevalent cancer type in the world and leads to 627,000 deaths per year [12]. It is considered the most common malignancy in women. Treatment methods depend on the molecular subtype and the breast cancer stage. The main treatments are surgery, chemotherapy, and hormonal therapies. The principal disadvantage of these treatments is their poor specificity for cancer cells because they attack or released factors that can damage the DNA of normal cells, affecting even more so the patient and causing collateral and irreversible damage in some cases [13]. The scientific community remains in the constant struggle to generate new strategies to avoid these side effects. i.e., the creation of drugs that directly attack cancer cells without damaging other tissues implying a lower risk of negative effects and improving the quality of life of the affected people. Even though the parasporin definition is: “*Bacillus thuringiensis* and related bacterial parasporal proteins that are non-hemolytic but capable of preferentially killing cancer cells” [4], a few reports are found in the literature about the effect of the parasporins against normal cells [5,8]. Therefore, the aim was to study a parasporin produced by *B. thuringiensis* that preferentially kills breast cancer cells and without toxicity against normal cells, as well as study the oxidative stress.

## 2. Results

### 2.1. Proteins Isolation

**Figure 1:**
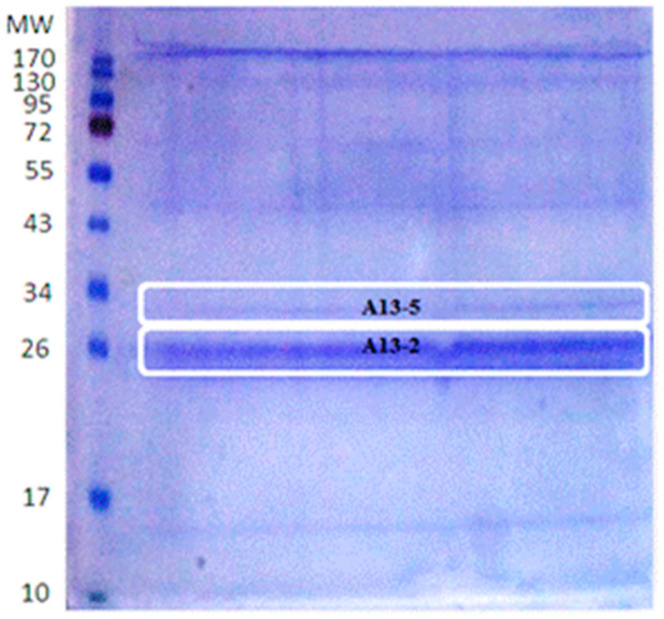
SDS-PAGE profile of parasporal inclusion proteins of *B. thuringiensis* isolate of A13 strain. Line 1, molecular weight marker, lane 2 solubilized parasporal inclusions of the isolate A13 strain.

Every *B. thuringiensis* strain produces different parasporal proteins and in different quantities. For *B. thuringiensis* A13, isolated from the Papaloapan region, polyacrylamide gel analysis shows that the strain majorly produces two parasporal proteins. After purification, the bands for cytotoxicity assay were selected according to their size and intensity; and they were called A13-2 (26 kDa) and A13-5 (30 kDa) (Figure 1).

### 2.2. Cytotoxic Effect of Parasporal Protein A13-2 in Cancer and PBMC Cells

**Figure 2:**
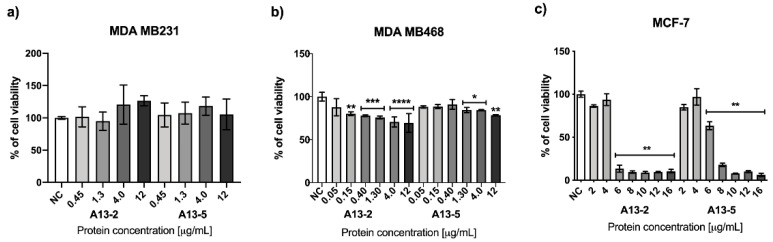
Cytotoxicity of *B. thuringiensis* parasporal inclusions against breast cancer cell lines; (**a**) MDA-MB 231, (**b**) MDA-MB 468, and (**c**) MCF-7 at 48 h of incubation. Results are expressed in percentage of negative control (NC). The data are mean ± standard deviation (SD). *p* < 0.05 were considered statistically significant. * represents *p* ≤ 0.05; ** *p* ≤ 0.01; *** *p* ≤ 0.001 and **** *p* ≤ 0.0001 all of them vs. negative control.

The cytotoxic activity assay results of the MDA-MB 231 and MDA-MB 468 cells treated with the parasporal proteins A13-2 and A13-5 during 48 h are shown in Figure 2a,b. The parasporal proteins do not affect MDA-MB 231 viability (Figure 2a). Parasporal protein A13-2 reduce the MDA-MB 468 viability by 30% at 4 μg/mL and protein A13-5 by 16% at 1.3 and 4 μg/mL (Figure 2b). MCF-7 cells treated with the parasporal protein A13-2 during 48 h are shown in Figure 2c. Data show that the A13-2 protein is cytotoxic against MCF-7, at 6 μg/mL cell viability has been reduced by 87%, while A13-5 protein at 8 μg/mL reduces cell viability by 80% (Figure 2c).

**Figure 3:**
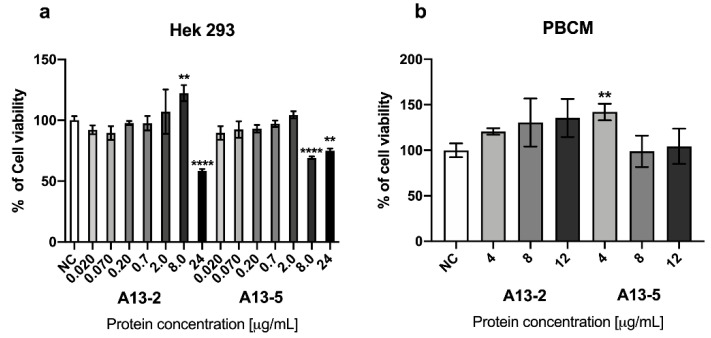
Cytotoxicity of *B. thuringiensis* parasporal inclusions against normal cells; (**a**) PBMC and (**b**) HEK 293 at 48 h of incubation. Results are expressed in percentage of negative control (NC). The data are mean ± standard deviation (SD). *p* < 0.05 were considered statistically significant. ** represents *p* ≤ 0.01 and **** *p* ≤ 0.0001 both vs. negative control.

MTT assays in normal cells PBMC and HEK 293 at 48 h incubation using the A13-2 and A13-5 treatments show no cytotoxic effect at the concentrations tested (Figure 3a,b). However, A13-5 protein against HEK 293 at 6 μg/mL cell viability has been increased by 17% (Figure 3b). MTT assays results (Figure 2 and Figure 3) suggests high selectivity of A13-2 and A13-5 parasporal proteins against cancerous cells. While the normal cells PBMC and HEK 293 treated with A13-2 and A13-5 showed no significant difference with the control. Also, these results indicate that MCF-7 cells are more susceptible to A13-2 protein than A13-5 and normal cells. Therefore, A13-2 protein was chosen for further analysis.

### 2.3. Role of Oxidative Stress in the Cytotoxicity of A13-2 Protein in MCF-7 and PBMC Cells

Cancer is an inducing oxidative stress disease. Metabolic changes in neoplastic cells, tumor infiltration by inflammatory cells, malnutrition, and specific cancer treatment contribute to high levels of oxidative stress in cancer patients [14].

**Figure 4:**
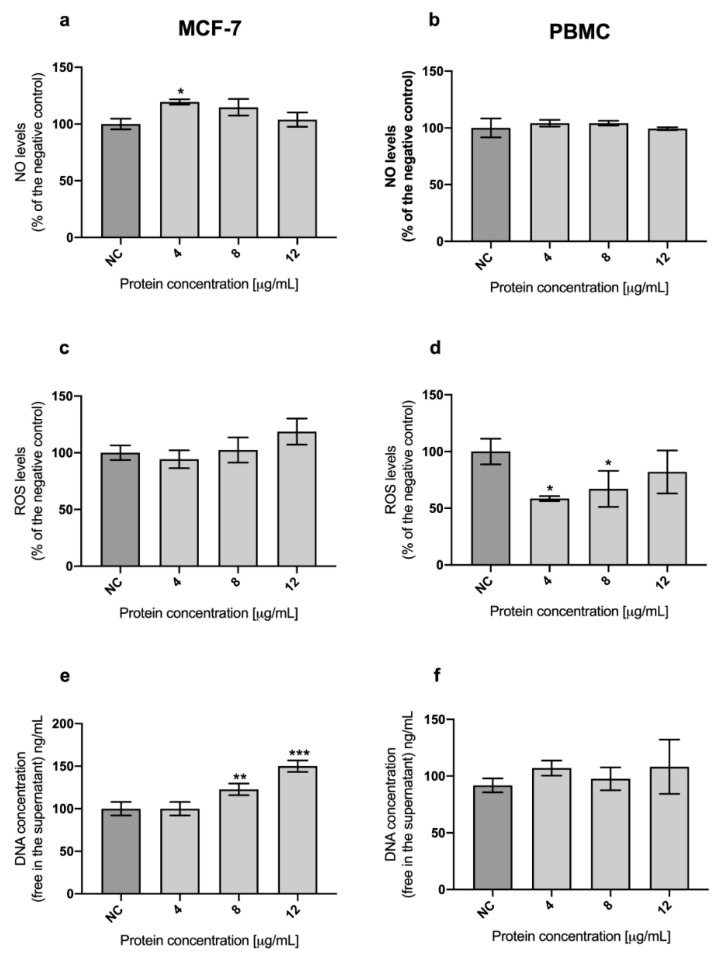
Oxidative stress role after treatment with *B. thuringiensis* parasporal protein A13-2 against MCF-7 cells at 72 h and normal cells (PBMC) at 48 h of incubation. Nitric oxide levels in MCF-7 (**a**) and PBMC (**b**). ROS levels in MCF-7 (**c**) and PBMC (**d**). Determination of DNA fragmentation in MCF-7 (**e**) and PBMC (**f**). Results expressed in percentage of negative control (NC). The data are mean ± SD. *p* < 0.05 were considered statistically significant. * represents *p* < 0.05, ** represent *p* < 0.01 and *** represent *p* < 0.001 vs. control.

Figure 4a,b show the results for Nitric Oxide (NO) assay of the parasporal protein against MCF-7 and PBMC, respectively. The parasporal protein treatments increase the NO levels to 24% at 4 μg/mL in MCF-7 cultures. That increment is not enough to trigger oxidative stress. There are no significant differences in NO levels for parasporal protein treatments against PBMC cells.

On the other hand, the levels of reactive oxygen species (ROS) after parasporal protein A13-2 treatments were not significantly different after 48 h in MCF-7 (Figure 4c); while in PBMC, a significant reduction in ROS levels were observed at concentrations of 4 and 8 µg/mL (Figure 4d).

The Picogreen technique was used to measure the free DNA in the supernatant after the treatment with A13-2 for MCF-7 and PBMC cells (Figure 4e,f). This technique indirectly evaluated the damage in the cells. The results for MCF-7 cells treated with parasporal protein A13-2 show an increment of DNA in the medium, and it is protein concentration-dependent (Figure 4e). This result confirms that A13-2 damages the cell in some way that the DNA could be detected in the supernatant. For PBMC treated with A13-2 there are no significant differences with the control (Figure 4f).

### 2.4. Morphological Changes and Cell Death Mechanism Induced by Parasporal Protein A13-2

**Figure 5:**
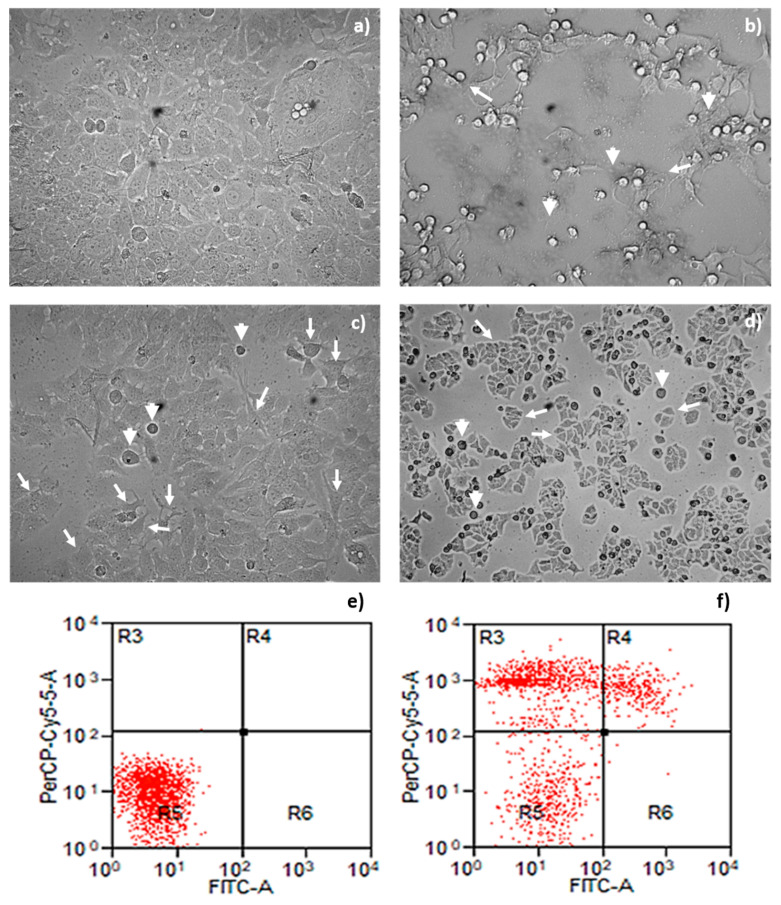
Cytopathic effect and apoptosis—necrosis determination of treatment with A13-2 protein to 24 h of incubation on MCF-7 cells. Cytopathic effect of protein of *B. thuringiensis* A13-2 on MCF-7, Negative control (**a**), Doxorubicin as positive control (**b**), A13-2 4 μg/mL (**c**) and A13-2 8 μg/mL (**d**). Arrowheads point out to apoptotic bodies while white arrows show cells with irregular morphology that are not typical of MCF-7 cells. Negative control cells (**e**); Annexin V-PI flow cytometry where the R3 expresses necrotic cells, R4 late apoptotic cells, R5 viable cells, and R6 early apoptotic cells after A13-2 protein 5 μg/mL (**f**). The inverted microscopic observations were done at 48 h post-inoculation. A mixture of 200 µL of DMEM/PBS was used as a negative control (NC).

Cell damage in cells is associated with morphological changes; thus, a microscopy analysis of MCF-7 cells with parasporal protein A13-2 treatment was performed (Figure 5). The cytopathic effect results are shown in Figure 5c–f. No detectable cytopathy was induced by A13-2 protein in the control group (Figure 5c). The cells show a normal angular or polygonal shape. At the same time, the cytopathy induced by the parasporal protein A13-2 an exhibited typical irregular morphology of cells in apoptotic processes on MCF-7 cells at 48 h of incubation (Figure 5e,f). The cells show as a round or oval dark mass associate with nuclear chromatin fragments; the morphology is almost normal polygonal shape with marked lesions that appear on the surface of the plasma membrane, indicating a late stage of apoptosis cell death (Figure 5e,f). As expected, doxorubicin evidence signs of apoptosis as the cytoplasm and chromatin condensation and the formation of apoptotic bodies (Figure 5d).

The apoptotic morphology of MCF-7 treated with A13-2 is confirmed with the cytometry test (Figure 5b). Annexin V and propidium iodide were used to determine if death was caused by necrosis or apoptosis. Most events are restricted to quadrant R5 in control cells, i.e., viable cells (Figure 5a). Cells treated with protein A13-2 have many events in quadrant R4 and R3 representing late apoptosis and necrosis, respectively (Figure 5b), suggesting that parasporal protein A13-2 induces high toxicity on MCF-7 cells. This result agrees with the MTT, NO, ROS and Picogreen test results.

## 3. Discussion

*B. thuringiensis* may produce different parasporins with different molecular weights and levels of cytotoxicity to diverse cancer cell lines. Nair et al. [15] analyzed 18 isolated strains of *B. thuringiensis* cultivated in T3 agar plates, incubated at 30 °C for 96 h, observing that at least six different parasporal proteins were produced by each isolated in different sizes and concentration. Brasseur et al. [16] reported that *B. thuringiensis* 4R2, cultivated at 30 °C on nutrient agar at pH 7.1, produces five parasporal proteins where the one at 37 kDa was identified as PS2Aa1. This variation in the number of parasporal proteins found is due to the growth and environmental conditions of each *B. thuringiensis* strain. In soil, the bacteria population is not randomly distributed because factors such as soil composition, organic matter, pH, water availability, and oxygen, along with the host plant, play an essential role in adapting this microflora [17].

MDA-MB-468 is a triple-negative breast cancer cell line. This breast cancer subtype is more aggressive than other forms, has a more unfavorable prognosis, and is predominantly treated with chemotherapy [18]. It is named triple-negative because it lacks estrogen receptor, progesterone receptor, and amplification of HER-2/Neu molecular markers [18]. Therefore, since A13-2 decreases 30% of the viability of MDA-MB-468 this protein could be studied in combination with other molecules or drugs to treat triple-negative breast cancer. There are no other reports about viability studies of a parasporal protein against MDA-MB-468 cells to the best of our knowledge.

The cytotoxicity effect of A13-2 and A13-5 (26 and 30 kDa, respectively) on MCF-7 cells was similar to that described in previous reports. Brasseur et al. [17] reported that PS2Aa1 (37 kDa) had good activity against MCF-7 at 2.5 µg/mL, reducing viability to 20%. However, the authors used chemical or enzymatic processes for protein activation. Maher [19] identified two strains J61 and J72, with cytotoxic activity against MCF-7 that reduce to 50% cell viability at 1 µg/mL and 2.79 µg/mL, respectively. Other authors tested differents parasporins observing no significant cytotoxic activity against MCF-7 [20,21,22]. It has been reported that a parasporin could affect more than one cancer line of cells. Brasseur et al. [16] and Maher [19] demonstrated that their parasporins also showed cytotoxicity against other cancer cells such as MDA-MB231, PC-3, HepG2, CACO-2, K562, and Hep2.

A13-2 and A13-5 treatments show no cytotoxic effect against normal cells (HEK and PBMC) at the concentrations tested (Figure 2). This result suggests that these parasporal proteins kill preferentially the cancerous cells tested and that they have potential to be used in cancer treatment. Moreover, considering that the A13-2 protein does not induce hemolysis (Figure S1) and that it kills preferencialy cancer cells therefore it could be named parasporin. A few studies have detected low or no activity in MTT assays of parasporin against normal immune cells and human embryonal kidney cells [20,23,24].

To mechanistically understand how parasporal proteins A13-2 reduces cell proliferation and to identify if oxidative stress is involved, NO and ROS levels were examined after exposure to different concentrations of parasporal protein A13-2. Cancer cells have a dual cellular response to oxidative stress. When the stress levels are low, the cells activate essential processes that guarantee their survival, while at high-stress levels, the cells died due to oxidative damage [25]. Moreover, cancer cells are more susceptibles to oxidative stress than normal cells; a small increment in the oxidative environment activates the death mechanisms in cancer cells but not in the normal cells [26].

The role of NO in cancer is complex, it has been reported that it is involved in multiple steps of tumor development, but it is also involved in the cell cycle arrest, i.e., in apoptosis. [27]. NO can act as an intra- and extracellular signaler participating in numerous pathological processes. It is a modulator in several essential biological processes, and it has a dubious role in cells, sometimes beneficial and sometimes harmful [28]. Also, NO is an important cytotoxic mediator of effector cells capable of destroying pathogens and tumor cells [29]. However, NO is potentially toxic, especially in situations of oxidative stress, generation of oxygen intermediates, and antioxidant system deficiencies [30]. The nitric oxide synthase (iNOS) is responsible for the endogenous production of NO. iNOS is only expressed when cytokines or endotoxins induce it in cells such as macrophages, T lymphocytes, neutrophils, and platelets, among others [31]. Hence, for MCF-7 and PBMC cultures with a parasporal protein treatment, the NO levels measured indicate that the cells were not induced to produce proinflammatory cytokines and that the parasporal proteins were not recognized as endotoxins.

The dichlorofluorescein (DCFH-DA) assay is the most widely used probe for detecting oxidative stress through measurement of ROS formation due to the increment in H_2_O_2_, due to the changes in intracellular iron signaling, and due to the peroxynitrite formation [32]. Natural ROS scavengers in MCF-7 and PBMC cells, like the glutathione peroxidase, catalase, and superoxide dismutase, can control the increment in the ROS levels and therefore maintain it in non-toxic levels. Likewise, the increase in PBMC proliferation observed after the A13-2 treatment might involve signaling mechanisms related to ROS levels. Previous studies described that low ROS levels in some blood cells induce its proliferation [33,34]. Most chemotherapeutic agents and radiotherapy treatment lead to apoptosis death cells by inducing intracellular ROS production [35,36]. Furthermore, it has been proposed that in peptide therapy, one possible mechanism of cell damage is associated with the increment of intracellular ROS [35,36]. Although, the results of the oxidative stress point out that the cytotoxic effect of parasporal protein A13-2 is not through this process.

Due to endogenous and exogenous factors, the DNA could be fragmented, and it could be exported out of the cells. Hence, through the detection of DNA in the medium, a genotoxic effect may be evaluated [37] and indirectly the formation of pores in the membrane [38]. Some authors proposed that one of the possible cytotoxic mechanisms of parasporins is through the formation of pores in the membrane of the tumor cells, which leads to an osmotic imbalance and consequently to cell death [39,40,41]. The Picogreen assay is an easy and fast technique to detect a minimum amount of dsDNA (25 pg/mL) in the medium [42]. Therefore, DNA in the medium was measured in MCF-7 cells after parasporal protein A13-2 treatment with Picogreen assay. The results for MCF-7 cells treated with parasporal protein A13-2 show that the increment of the double-stranded DNA (dsDNA) in the medium is protein concentration-dependent (Figure 4e). The dsDNA fragments release was significant at 8 and 12 µg/mL, indicating that MCF-7 cells were damaged, probably due to cellular apoptosis and necrotic cell (Figure 4e). Although, in PBMC cells, this damage was not observed (Figure 4f). Currently, there is no data available in the literature about the ROS and NO levels or even in the fragmented dsDNA concentration in cultures treated with parasporin. However, what is clear is that there is specific membrane damage induced by parasporal protein A13-2 on the breast cancer cells, where the formation of pores in the membrane might be related.

### Morphological Changes and Cell Death Mechanism Induced by Parasporal Protein A13-2

The MTT test shows that A13-2 promotes cell death, and NO-ROS results show that this protein does not trigger oxidative stress in MCF-7 (Figure 4a–d). Moreover, the Picogreen test shows fragmented free dsDNA in the medium associated with death by necrosis at 48 h (Figure 4e). Additionally, cytometry test indicated that at 48 h with A13-2, most of the cells were death by necrosis; however, a small percentage were at late apoptosis, suggesting a rapid transition from late apoptosis to necrosis, which could point out that lower concentrations or times-course analysis perhaps will allow us to observe more apoptotic signals. Therefore, A13-2 parasporin leads to the death of MCF-7 cells by late apoptosis and necrosis and does not trigger the survival mechanisms.

It has been reported different mechanisms of cell death treated with parasporins. PS1Aa1 against HeLa cells showed a rapid intracellular increment of Ca^2+^ but without lactate dehydrogenase release and IP internalization, which is strong evidence of death by apoptosis [43]. For PS2Aa1 against HepG2, an increment in the membrane permeability was observed where the lactate dehydrogenase extravasation and the PI internalization are due to a similar mechanism to *Clostridium perfringens* epsilon toxin. PS2Aa1 has homology with epsilon toxin, a pore-forming when it is in contact with lipid rafts [44]. PS3Aa1 has closely resembled pore-forming insecticidal Cry proteins, where cancer cells die by increasing membrane permeability [45]. PS4 is nonspecifically bound to the membrane forming a cholesterol-independent oligomeric pore complex and shows some homology to α-toxin, aerolysin, and ε-toxin [46]. PS5 has no similarity to other parasporins or Cry proteins but exhibits some homology to β-pore-forming aerolysins (β-TFPs) and epsilon toxin that is a pore-forming toxin [47]. There is little information about PS6. However, it is considered a three-domain Cry protein with 56.4% identity to Cry2 insecticidal proteins [46]. Thus, all our results show that A13-2 is a parasporin with a cytotoxic effect over MCF-7 cells that could interact with the cell membrane, leading to the formation of pores that drive cell death by late apoptosis with necrosis. However, more experiments must be done to clarify if their mechanism is like PS2, PS3, PS4, or PS5.

## 4. Conclusions

The main disadvantage of existing cancer therapies is that they are also toxic to normal cells, generating several side effects and deteriorating the patient’s quality of life. The parasporin produced for *B. thuringiensis* A13-2 has a cytotoxic effect on MCF-7 cancer cells. It does not have toxic activity against peripheral blood mononuclear cells (non-cancerous cells), i.e., it is specific for the cancer cells. Also, this parasporin leads to cell death by late apoptosis without triggering oxidative stress. The fundamental goal of cancer treatments is to identify molecules with a high capacity to induce selective cancer cell death, blocking any chance of activating the survival mechanisms of the cancer cells. In this context, late apoptosis induced by parasporal protein A13-2 together with necrosis could be the best death mechanism, as both mechanisms are irreversible for cells. It has been reported that cancer cells can progressively develop acquired resistance to apoptotic cell death; thus, with parasporal protein A13-2, cell death occurs quickly without allowing the triggering of the survival mechanisms. Large-scale production of *B. thuringiensis* is well established as well as the extraction of their crystalline proteins; therefore its production by the pharmaceutical industry is plausible and perfectly executable. Although further studies regarding the mechanisms of cell death and in vivo effects of these proteins have to be done. Moreover, about the elucidation of protein structure, the pathways of cytotoxicity and non-toxicity towards normal cells could lead, in the future, to the drug design.

## 5. Materials and Methods

### 5.1. Bacterial Strains, Culture Conditions and Parasporal Inclusion Isolation

*B. thuringiensis* strains used were isolated from the Papaloapan watershed region [47]. The strains were cultivated in a Gerry-Rowe medium (MCD Lab.) at a pH of 7.4 (adjusted with 40% NaOH) at 180 rpm for seven days at 30 °C (New Brunswick Scientific Benchtop Incubator Shaker I24, Eppendorf, New Brunswick, Canada) to obtain the protein extracts. Once the fermentation was concluded, the culture medium was centrifuged at 5500 rpm for 20 min at 5 °C and washed with distilled water, acidified water (pH 2.5; three times), distilled water, NaCl 0.85% *w/v* (three times), and distilled water, in that order. For every wash, the pellet was resuspended of the solution and centrifuged at 5500 rpm for 20 min at 5 °C. After washing, the pellet was resuspended in 5 mL of distilled water [10].

All the washed protein extracts were solubilized with 1:2 of Laemmli buffer at 100 °C for 5 min [48] for parasporal inclusions isolation. Then, the protein extracts were separated by molecular weight through sodium dodecyl sulfate-polyacrylamide gel electrophoresis (SDS-PAGE) at 12%, with bovine serum albumin (BSA) as a reference protein. The molecular masses of proteins were estimated by molecular mass standards (170, 130, 95, 72, 55, 43, 34, 26, 17, and 10 kDa, ThermoScientific, Wilmington, NC, USA). The interest bands (32 to 26 kDa) were cut from the gel and recovered by electroelution at 10 mA for 210 min in a Mini-Protean^®^ system (Bio-Rad, Hercules, CA, USA). The eluted proteins were dialyzed using bioseparation columns (Ultrafree-MC for volumes less than 0.5 mL with a pore size of 6000 Da) to remove the SDS reagent and obtain parasporal inclusions-SDS free. Washes were carried out with phosphate buffer (PBS) at 13,300 rpm, 10 min at 4 °C. Protein quantification was done in a NanoDrop 2000/2000c instrument (ThermoScientific, Wilmington, NC, USA) using the Protein A280 method.

### 5.2. Cell Culture Conditions

Peripheral blood mononuclear cells (PBMCs; non-cancerous cells) were donated from the Clinical Analysis Laboratory of the Franciscan University (LEAC-UFN). The LEAC-UFN laboratory obtained the cells from blood samples discarded from healthy adults (experimental protocols used were approved by the UFN Ethics Committee on Human Beings; CAAE number: 31211214.4.0000.5306) with the absence of identification data. PBMC was maintained in Roswell Park Memorial Institute Medium (RPMI 1640 Biowest, Riverside, CA, USA) at pH 7.4, supplemented with 10% fetal bovine serum (Biowest), 100 U/mL penicillin, 100 μg/mL streptomycin (Biowest), and 2 mM L-glutamine (Biowest). The PBMC was maintained at 37 °C with saturated humidity and 5% CO_2_. All procedures performed were following the ethical standards of the institutional and/or national research committee and with the 1964 Helsinki declaration. According to the UFN Ethics Committee on Human Beings, signed informed consent was no needed.

The non-cancerous cells HEK 293 (human embryonal kidney cells) and the human breast cancer cell line MDA MB 231, MDA-MB 468, and MCF-7 were acquired from American Type Culture Collection (ATCC, Manassas, VA, USA; ATCC^®^ CRL-1573™; ATCC^®^ HTB-26™; ATCC^®^ HTB-132™; ATCC^®^ HTB-22™; respectively). The non-cancerous cells HEK 293 and the human breast cancer cell lines were at passage 12 when it was used in the study and routinely cultured on monolayers at 80% confluence in Dulbecco’s modified Eagle’s High glucose medium (DMEM, Biowest), supplemented with 10% fetal bovine serum (Biowest), 100 U/mL penicillin, 100 μg/mL streptomycin (Biowest), and 2 mM L-glutamine (Biowest). All line cells were maintained at 37 °C with saturated humidity and 5% CO_2_. The growth medium was removed to harvest the human breast cancer cell lines, and cells were washed with phosphate buffer saline (PBS, Biowest). A cell dissociation solution made of trypsin-EDTA (Biowest) was added and incubated at 37 °C for 3 min in a humidified 5% CO2 incubator to produce a cellular suspension. Trypsinized cells were reseeded in a fresh medium at 10^5^ cells/mL and incubated at 37 °C in a humidified 5% CO_2_ incubator.

### 5.3. Cytotoxicity Assays

The cytotoxicity of parasporal inclusions was evaluated by 3-(4,5-dimethylthiazol-2-yl)-2,5 diphenyltetrazolium bromide (MTT) assay [49]. MCF-7 and PBMC cells (2500 cell/20 μL) were cultured in 96-well microplates with RPMI 1640 medium and DMEM high glucose, respectively, and incubated at 5% CO_2_ for 24, 48, or 72 h with 4 to 16 μg/mL of protein. After incubation, 20 μL of MTT (5 mg/mL) dissolved in PBS were added and incubated for 4 h. At the end of this time, the medium was removed, and the insoluble formazan crystals were dissolved with 200 μL of dimethylsulfoxide (DMSO). The optical density produced by this product was read at a wavelength of 595 nm using a microplate reader (Bio-Rad iMark). Each treatment was performed by triplicate and repeated three times using 200 µL of DMEM as a negative control (NC).

### 5.4. Nitric Oxide Test

Nitric Oxide (NO) production was evaluated as metabolite involved in the apoptosis induction for MCF-7 and PBMC cells [50,51]. After 72 and 48 h incubation of MCF-7 and PBMC, respectively, the culture plate was centrifuged 10 min at 2000 rpm with the parasporal protein treatments. In a new plate, 50 μL of the supernatant and 50 μL of the Griess reagent (1% sulfanilamide and *N*-1-naphthylethylenediamine dihydrochloride 0.1%) were added. The plate was incubated for 15 min at room temperature. Subsequently, the absorbance produced was read in a TP-Reader plate reader Thermoplate (Bio Tek instruments, Winooski, VT, USA) at 570 nm [52,53]. 200 µL of DMEM was used as a negative control (NC). The data were expressed as a percentage of free NO in the medium concerning the negative control:

% NO free in the middle = (Absorbance of the sample * 100)/average of the negative control.

### 5.5. Reactive Oxygen Species Assay

Dichlorofluorescein (DCFH-DA) was used to indirectly measure the total rate of reactive oxygen species (ROS) present. After 72 and 48 h incubation of MCF-7 and PBMC, respectively, with the parasporal treatments, the culture plate was centrifuged for 10 min at 2000 rpm, and in a dark new plate was added 100 μL of supernatant, 130 μL of Tris-HCl (10 mM, pH 7.4) and 20 μL of DCFH-DA (1 mM). 200 µL of DMEM was used as a negative control. The plate was incubated for 60 min in the dark at room temperature. The reading was done on a fluorescence meter (SpectraMax^®^ i3x -Molecular Devices, San Jose, CA, USA) at 525 nm emission wavelength and 488 nm of excitation [54,55]. The data were expressed as a percentage of the total rate of ROS concerning the negative control:

% Total rate of ROS = (Absorbance of the sample * 100)/average of the negative control.

### 5.6. Genotoxicity Assay

The Picogreen test is fluorimetric and quantifies the double strand of DNA by binding to it and emitting fluorescence when released into the medium by cellular apoptosis or necrosis. After 72 and 48 h incubation of MCF-7 and PBMC, respectively, the culture plate was centrifuged for 10 min at 2000 rpm with parasporal protein treatments. 10 μL of the supernatant was placed in a new dark plate, homogenized with 80 μL of TE buffer, and it was added 10 μL of the Picogreen reagent diluted in TE buffer (1:200). The reaction was incubated at room temperature and in the darkness for 5 min. The reading was performed on a fluorimeter (SpectraMax^®^ i3x -Molecular Devices) at a 520 nm emission wavelength and 480 nm of excitation [56], and 200 µL of DMEM was used as a negative control. The data were expressed as a percentage of a double strand of free DNA in the medium concerning the negative control:

% Dc of free DNA in the medium = (Fluorescence * 100)/mean of the negative control.

### 5.7. Apoptosis Detection by Annexin V/PI Assay

FITC Annexin V/Dead Cell Apoptosis Kit (Molecular Probes Inc., Eugene, OR, USA) was used according to the manufacturer’s instructions. Annexin V binds to phosphatidylserine on the outer leaflet of the plasma membrane, and its presence on the outer leaflet is a unique feature of early apoptosis. Propidium iodide (PI) binds to DNA from cells with the disrupted cell membrane, as in late apoptosis and necrosis, being excluded from cells with intact membrane [57]. Cells incubated 24 h with parasporal protein were collected, washed with PBS, and diluted in 1X Annexin Binding buffer (100 μL). For each sample, 5 μL of Annexin V and 2 μL of PI were added to the cell suspension and incubated for 15 min at room temperature. An additional 100 μL volume of Annexin binding buffer was added to each sample for a total of 200 μL. Samples were analyzed (5000 events) using a BD FACSAria flow cytometer, and the analysis was performed using BD FACSDiva software (Becton, Dickinson and Company, Franklin Lakes, NJ, USA). A mixture of 200 µL of DMEM/PBS was used as a negative control (NC).

### 5.8. Light Microscopic Observation

The possible MCF-7 cell morphological changes induced by parasporal protein A13-2 incubated during 48 h were analyzed by light microscopy with a 20× objective and 10× ocular lenses. The cytopathic effect was monitored by an inverted microscope (Motic AE31E, Moticam 5 plus, MOTIC, Kowloon, Hong Kong, China). The images were captured and analyzed for cell morphology with the Motic images plus 3.0 software. A mixture of 200 µL of DMEM/PBS was used as a negative control (NC).

### 5.9. Statistical Analysis

Data were analyzed using a One-Way ANOVA (Analysis of Variance) with GraphPad Prism software version 5.0 (GraphPad Software, Inc., San Diego, CA, USA). The Dunnet test was applied to compare each treatment with the control, with a statistical significance of *p* < 0.05.

## Data Availability

Not applicable.

## Supplementary Materials

The following are available online at https://www.mdpi.com/article/10.3390/toxins13070476/s1, Figure S1: Hemolysis assay of A13-2 protein. 10 μL of 20% Triton X-100 was used as a positive control (PC). The assay was conducted whit Evans et al. (2013) methodology using 190 μL diluted red blood and 10 μL of A13-2 protein dilutions.
