# Supplementary material for: Parasporin A13-2 of Bacillus thuringiensis Isolates from the Papaloapan Region (Mexico) Induce a Cytotoxic Effect by Late Apoptosis against Breast Cancer Cells
Source: Toxins (Basel). 2021 Jul 9;13(7):476. doi: 10.3390/toxins13070476 (PMC8309972; doi:10.3390/toxins13070476)
Supplement: Supplementary file 1 [file toxins-13-00476-s001.zip › toxins-1219831-supplementary.pdf]

Supplementary Materials: Parasporin A13-2 of *Bacillus thuringiensis* Isolates from the Papaloapan Region (Mexico) Induce a Cytotoxic Effect by Late Apoptosis against Breast Cancer Cells

Diego Becker Borin, Karen Castrejón-Arroyo, Alain Cruz-Nolasco, Miguel Peña-Rico, Michele Sagrillo Rorato, Roberto C. V. Santos, Lucas Silva de Baco, Lemuel Pérez-Picaso, Luz Camacho and A. Karin Navarro-Mtz

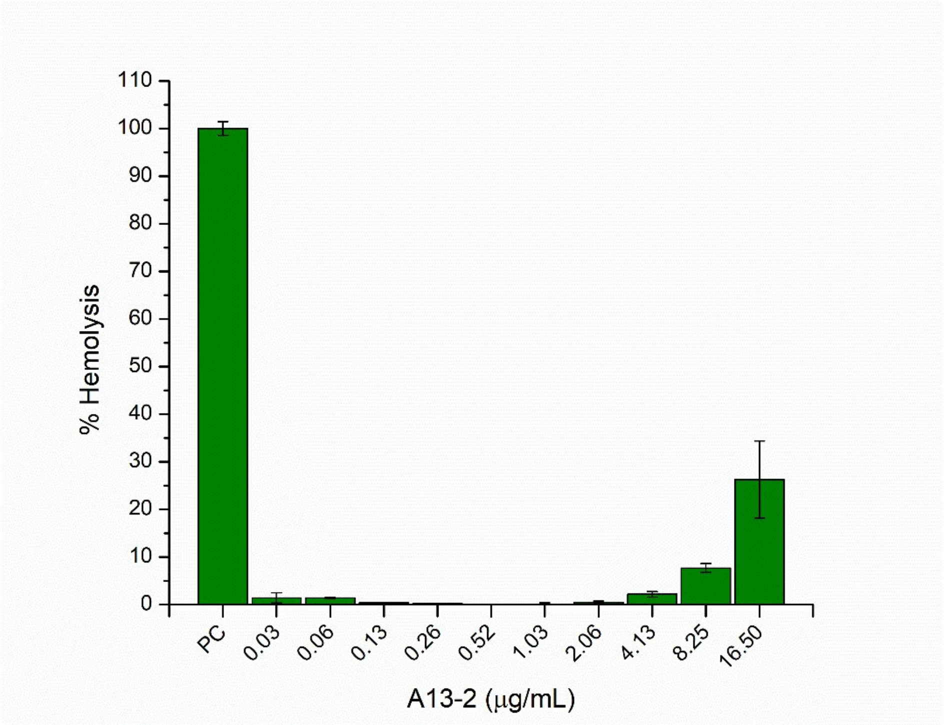

Figure S1. Hemolysis assay of A13-2 protein. 10 µL of 20% Triton X-100 was used as a positive control (PC). The assay was conducted with Evans et al. (2013) methodology using 190 µL diluted red blood and 10 µL of A13-2 protein dilutions.

[Evans BC, Nelson CE, Yu SS, Beavers KR, Kim AJ, Li H, Nelson HM, Giorgio TD, Duvall CL. Ex vivo red blood cell hemolysis assay to evaluate pH-responsive endosomolytic agents for cytosolic delivery of biomacromolecular drugs. *J Vis Exp*. **2013** 9, e50166. doi: 10.3791/50166. PMID: 23524982; PMCID: PMC3626231].
